# Prognostic value of the tumor-to-liver density ratio in patients with metastatic colorectal cancer treated with bevacizumab-based chemotherapy. A post-hoc study of the STIC-AVASTIN trial

**DOI:** 10.1186/s40644-024-00722-7

**Published:** 2024-06-17

**Authors:** Thibault Mazard, Caroline Mollevi, Evelyne M. Loyer, Julie Léger, Romain Chautard, Olivier Bouché, Christophe Borg, Paul Armand-Dujardin, Aurore Bleuzen, Eric Assenat, Thierry Lecomte

**Affiliations:** 1 Medical Oncology Department, Montpellier Cancer Institute (ICM), University of Montpellier, Institut de Recherche en Cancérologie de Montpellier (IRCM), INSERM U1194, 208 avenue des apothicaires, Parc Euromédecine, Montpellier Cedex 5, Montpellier, 34298 France; 2 Institute Desbrest of Epidemiology and Public Health, University of Montpellier, INSERM, Cancer Institute of Montpellier, Montpellier, France; 3 Department of Diagnostic Radiology, The University of Texas MD Anderson Cancer Center, Houston, TX USA; 4 INSERM CIC 1415, CHRU de Tours, Tours Cedex 9, 37044 France; 5 Department of Hepatogastroenterology and Digestive Oncology, UMR INSERM U 1069, Hôpital Trousseau, CHRU de Tours, Université de Tours, Tours Cedex 9, 37044 France; 6 Department of Hepatogastroenterology, Hôpital Robert Debré, CHU de Reims, Avenue Général Koenig, Reims Cedex, 51092 France; 7 Department of Medical Oncology, Hôpital Jean Minjoz, CHRU de Besançon, 3 Boulevard Alexandre Fleming, Besançon, 25000 France; 8 CHRU Tours, Inserm, CIC 1415, Tours Cedex 9, 37044 France; 9 Department of Radiology, CHRU de Tours, Tours Cedex 9, 37044 France; 10 Medical Oncology Department, Montpellier Cancer Institute (ICM), University of Montpellier, CHU Montpellier, Montpellier, France

## Abstract

**Background:**

The Response Evaluation Criteria in Solid Tumors (RECIST) are often inadequate for the early assessment of the response to cancer therapy, particularly bevacizumab-based chemotherapy. In a first cohort of patients with colorectal cancer liver metastases (CRLM), we showed that variations of the tumor-to-liver density (TTLD) ratio and modified size-based criteria determined using computed tomography (CT) data at the first restaging were better prognostic criteria than the RECIST. The aims of this study were to confirm the relevance of these radiological biomarkers as early predictors of the long-term clinical outcome and to assess their correlation with contrast-enhanced ultrasound (CEUS) parameters in a new patient cohort.

**Methods:**

In this post-hoc study of the multicenter STIC-AVASTIN trial, we retrospectively reviewed CT data of patients with CRLM treated with bevacizumab-based regimens. We determined the size, density and TTLD ratio of target liver lesions at baseline and at the first restaging and also performed a morphologic evaluation according to the MD Anderson criteria. We assessed the correlation of these parameters with progression-free survival (PFS) and overall survival (OS) using the log-rank test and a Cox proportional hazard model. We also examined the association between TTLD ratio and quantitative CEUS parameters.

**Results:**

This analysis concerned 79 of the 137 patients included in the STIC-AVASTIN trial. PFS and OS were significantly longer in patients with tumor size reduction > 15% at first restaging, but were not correlated with TTLD ratio variations. However, PFS was longer in patients with TTLD ratio > 0.6 at baseline and first restaging than in those who did not reach this threshold. In the multivariate analysis, only baseline TTLD ratio > 0.6 was a significant survival predictor. TTLD ratio > 0.6 was associated with improved perfusion parameters.

**Conclusions:**

Although TTLD ratio variations did not correlate with the long-term clinical outcomes, TTLD absolute values remained a good predictor of survival at baseline and first restaging, and may reflect tumor microvascular features that might influence bevacizumab-based treatment efficiency.

**Trial registration:**

NCT00489697, registration number of the STIC-AVASTIN trial.

## Introduction

Colorectal cancer remains one of the most common and aggressive cancers in Europe, with approximately 500,000 new cases in 2018 [1]. Most patients will develop metastases that are often disseminated and unresectable [2]. The current guidelines recommend upfront systemic combination treatments with cytotoxic chemotherapy and biologic agents that inhibit mechanisms associated with cancer progression (e.g. angiogenesis inhibitors) [3–5]. Bevacizumab, the first approved antiangiogenic drug, is a monoclonal antibody that binds to and antagonizes vascular endothelial growth factor (VEGF) A, a key tumor angiogenesis factor. The combination of fluoropyrimidine-based chemotherapy plus oxaliplatin and/or irinotecan with bevacizumab as first- or second-line treatment for metastatic disease significantly improves patient outcomes in bevacizumab-naive patients [6–8]. The combination of bevacizumab and fluoropyrimidine also provides a survival benefit as maintenance therapy in patients with good response to a more intensive initial treatment, as demonstrated by the phase III CAIRO3 trial [9]. Furthermore, Bennouna et al*.* showed that bevacizumab continuation with a second-line chemotherapy regimen after the first progression prolonged overall survival (OS) compared to chemotherapy alone. This validated the concept of the “continuous anti-angiogenic blocking” approach [10]. However, although bevacizumab is now routinely used, there is no robust predictive marker to identify the patients who are more likely to benefit from angiogenesis inhibitors [11]. Moreover, bevacizumab efficacy is not always associated with tumor shrinkage [12]. The standard Response Evaluation Criteria in Solid Tumors (RECIST), based on tumor long axis measurements in axial computed tomography (CT) images, may be inadequate for the early assessment of bevacizumab efficacy. Therefore, alternative radiologic biomarkers have been investigated. Several studies using functional imaging modalities, such as contrast-enhanced ultrasound (CEUS), found a correlation between patient outcomes and early changes in liver tumor perfusion parameters in response to anti-VEGF pathway agents [13, 14]. Similarly, in patients with colorectal cancer liver metastases (CRLM) treated with bevacizumab before surgical resection, optimal morphologic response (*i.e.* the transformation into lesions with a homogeneous overall attenuation and a sharp tumor-liver interface), but not the RECIST, was associated with a better pathologic response and better outcomes [15]. The on-treatment early prognostic value of the morphologic response was subsequently confirmed in patients with unresectable CRLM [16–18].

Recently, we developed an alternative method based on tumor lesion size and attenuation assessment that can be easily performed using standard portal venous phase CT images. We showed that in CRLM, progression-free survival (PFS) and OS are significantly longer in patients with tumor size reduction > 15% and/or a tumor-to-liver density (TTLD) ratio variation not lower than -10% at the first restaging CT after the initiation of first-line combination therapy with XELIRI or FOLFIRI [19].

The aim of this post-hoc study was to confirm our first results in an independent population with CRLM treated with chemotherapy plus bevacizumab. We also evaluated the correlation between TTLD and CEUS parameters to better understand how bevacizumab provides its real benefit.

## Material and methods

### Study design

We performed a post-hoc analysis of contrast-enhanced CT (CECT) images from a prospectively accrued cohort of 137 patients with unresectable CRLM treated with bi-weekly bevacizumab-based chemotherapy as first-line therapy in a French multicenter noncomparative trial (NCT00489697, ID number INCA06-FT/STIC-AVASTIN) from January 2007 to November 2010. The objective of the main study was to assess the correlation between liver CEUS parameters and radiological response and outcome [14]. Patients underwent CT imaging with multidetector row helicoidal acquisitions before treatment and at day 60 after treatment start, then every 8 or 12 weeks at the investigator’s discretion.

### Patient selection

Patients with at least one liver metastasis larger than 1.5 cm, with baseline and restaging CT data of sufficient quality to allow assessing the density response, and with images acquired during the portal venous phase following intravenous contrast injection with a slice thickness < 3 mm were included in this post-hoc study. The contrast enhancement quality was assessed by analyzing portal and hepatic vein attenuation. All CECT images with a vascular density < 100 Hounsfield Units (HU) were excluded from the analysis.

### Image analysis

All CT images used in this study were anonymized. Target liver lesions on the pretreatment (baseline) CT were measured manually as well as the long axis diameters according to the RECIST 1.1. A maximum of two metastases were selected in patients with multiple tumor lesions. Then, volumetric segmentation of the tumor was performed using a semi-automated edge detection software (Myrian®, Intrasense, Montpellier, France). Tumor edges were adjusted until a satisfactory three-dimensional selection of a target lesion was obtained, and then the mean tumor volumetric attenuations were measured. The software also automatically segmented the healthy liver, excluding metastases and hepatic vessels, to calculate the mean density. To compensate for intra- and inter-individual heterogeneity in liver contrast enhancement, the TTLD ratio was determined, *i.e.* the mean tumor density divided by the mean healthy liver density. The same analysis was performed using CT images obtained at the first restaging. To minimize inter-observer variability, target liver lesion selection, size measurement and segmentation were performed by a single operator (T.M., GI oncologist) trained in the use of the software and involved in a previous study using this approach [19]. Whenever possible, the radiologist (E.L.) who contributed to develop the MD Anderson criteria [15] also assessed the morphologic response.

### CEUS parameters

When available, the relative values (i.e. compared to healthy liver, expressed in percentage) of 11 CEUS parameters determined at day 0 (D0) and day 60 (D60) were obtained from the main study: peak enhancement (PE), area under the curve during wash-in (WiAUC) and wash-out (WoAUC), total area under the curve (AUC = WiAUC + WoAUC), time-to-peak (TTP), rise time (RT), fall time (FT), wash-in (WiR) and wash-out (WoR) rates, wash-in perfusion index (WiPI), and mean transit time (mTT).

### Statistical analysis

Categorical variables were reported as numbers and frequencies. Continuous variables were reported as median and range and compared with the Wilcoxon rank-sum test.

The mean tumor density was evaluated using a threshold of 63 HU to stratify patients in accordance with the results by Dohan et al*.* [20]. The TTLD ratio variations were analyzed using a -10% cut-off, as established in our previous work [19]. Moreover, the median TTLD ratio at baseline was used as a categorizer. Modified RECIST (RECIST_-15_) were used for size reduction at the first restaging, with response defined as a > 15% reduction in the sum of target liver lesions.

PFS was the primary endpoint, defined as the time from the baseline CT exam to the date of the first disease progression or death from any cause. Patients alive without disease progression were censored at the date of the last visit. OS was the secondary endpoint, defined as the time from the baseline CT exam to death from any cause. The median follow-up was calculated using the Schemper method. PFS and OS were estimated using the Kaplan–Meier method and reported as median or rate at specific time points with their 95% confidence intervals (CI).

A Cox proportional hazard model was used to estimate the hazard ratios (HR) with their 95%CI for the prognostic factors associated with PFS and OS. Variables with p-values < 0.15 in the univariate analysis were retained for the multivariate analysis and a backward covariate selection was performed.

## Results

### Population

**Fig. 1:**
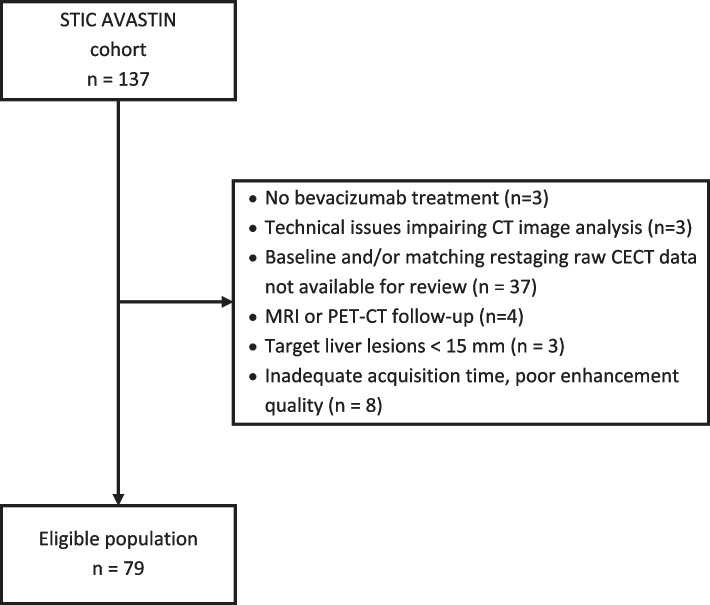
Study flowchart

In total, 79 of the 137 patients enrolled in the main trial were eligible for this post-hoc analysis. The reasons for exclusion are listed in Fig. 1. Their clinical characteristics and outcomes were similar to those of the whole population (data not shown). Table 1 shows the characteristics of the 79 patients.

**Table 1:** Patients’ characteristics

Patients’ characteristics (*n* = 79)	n (%)^a^
**Age** (years)	64 [38–82]
**Sex**	
Male	48 (61)
Female	31 (39)
**ECOG-PS score**	
0	37 (47)
1	40 (49)
**Primary tumor site**	
Colon	61 (71)
Rectum	25 (29)
**Metastases**	
Synchronous	61 (77)
Metachronous	18 (23)
**Chemotherapy regimen**	
FOLFIRI	56 (71)
FOLFOX	5 (6)
LV5FU2	7 (9)
Other	11 (14)
**LDH** (UI/L)	750 [111–3510]
**CEA** (ng/mL)	625 [1–6013]

*ECOG-PS* Eastern Cooperative Oncology Group—Performance Status, *LDH* Lactate dehydrogenase, *CEA* Carcinoembryonic antigen

^a^Except for age, LDH and CEA that are reported as medians [SD]

### Radiological parameters

The median (range) time from baseline CT to the first restaging CT was 2.3 (1.6–3.4) months. The median PFS and OS were 11 (95% CI: 9.4, 12.2) and 25.1 months (95% CI: 21.2, 31.8), respectively, and the median follow-up was 50 months (1.9–64.2). The baseline mean tumor density ranged from 23.9 to 117.3 HU and the median value was 62.8 HU. At the first restaging, the mean tumor density ranged from 6.9 to 179.5 HU and the median value was 53.5 HU. The median TTLD ratio was 0.62 at baseline and 0.54 at the first restaging (Table 2).

**Table 2:** Radiological parameters

Radiological parameters (*n* = 79)	Median [range]^a^
**Total diameter of the target lesions (mm)**	
*At baseline*	78.2 [14.7–199.3]
*At the first restaging*	62 [6.9–179.5]
**Mean tumor density (HU)**	
*At baseline*	62.8 [23.9–117.3]
*At the first restaging*	53.5 [6.9–179.5]
**Mean healthy liver density (HU)**	
*At baseline*	99.2 [27.6–134.8]
*At the first restaging*	99.8 [39.2–143.9]
**TTLD ratio**	
*At baseline*	0.62 [0.34–2.06]
*At the first restaging*	0.54 [0.28–1.14]
**RECIST**_**-15**_^b^	
CR/PR	50 (63)
SD	27 (34)
PD	2 (3)
**MD Anderson morphologic response**^b^	
Optimal	31 (47)
Suboptimal or no response	35 (53)
Missing	13

*TTLD* Tumor-to-liver density ratio, *RECIST*_*-15*_ Response Evaluation Criteria in Solid Tumors using a modified cut-off value of -15%, *CR* Complete response, *PR* Partial response, *SD* Stable disease, *PD* Progressive disease

^a^Except for the RECIST_-15_ response and MD Anderson response that are reported as n (%)

^b^Recorded at the first restaging

At the first restaging, according to the RECIST_-15_, 63%, 34%, and 3% of patients were classified as having a complete/partial response, stable disease, or progressive disease, respectively. Moreover, 31 (47%) patients achieved an optimal morphologic response, according to the MD Anderson morphologic criteria.

### Associations between quantitative CT parameters and survival

Four variables were identified as significant (p < 0.05) prognostic parameters of PFS in the univariate analysis: time of metastasis diagnosis (synchronous *vs.* metachronous; HR = 0.57, 95% CI: 0.32- 1.00), RECIST_-15_ value at the first restaging (≥ -15% *vs.* < -15%; HR = 1.77, 95% CI: 1.09–2.88), and TTLD ratio at baseline (> 0.6 *vs.* ≤ 0.6; HR = 0.53, 95% CI: 0.33–0.86) and first restaging (> 0.6 *vs.* ≤ 0.6; HR = 0.58, 95% CI: 0.35–0.96). Moreover, PFS tended to be shorter in patients with non-optimal morphologic response (2–3 *vs.* 1; HR = 1.44, 95% CI: 0.88–2.37). Therefore, this parameter was kept for the multivariate analysis. In the multivariate analysis, only baseline TTLD ratio (> 0.6 *vs.* ≤ 0.6; HR = 0.51, 95% CI: 0.29–0.88) and morphologic response according to the MD Anderson criteria (1 *vs.* 2–3; HR = 1.79, 95% CI: 1.05–3.05) remained significant (Table 3).

**Table 3:** Univariate and multivariate Cox models to identify associations between clinical/ radiological factors and PFS

	**Univariate analysis (*n* = 79)**		**Multivariate analysis (*n* = 66)**	
	**HR [95% CI]**	***p***	**HR [95% CI]**	***p***
**Age**		0.167		
< 60 years	1			
[60 years; 70 years]	0.72 [0.41–1.23]			
≥ 70 years	1.25 [0.69–2.27]			
**ECOG-PS score**		0.728		
0	1			
1	0.92 [0.58–1.47]			
**Primary tumor site**		0.188		
Colon	1			
Rectum	1.41 [0.86–2.33]			
**Time of metastasis diagnosis**		**0.046**		
Synchronous	1			
Metachronous	0.57 [0.32–1.00]			
**Mean tumor density**				
*At baseline*		0.144		
≤ 63 HU	1			
> 63 HU	0.70 [0.45–1.13]			
*At the first restaging*		0.238		
≤ 63 HU	1			
> 63 HU	0.74 [0.45–1.23]			
**TTLD ratio**				
*At baseline*		**0.010**		**0.016**
≤ 0.6	1		1	
> 0.6	0.53 [0.33–0.86]		0.51 [0.29–0.88]	
*At the first restaging*		**0.029**		
≤ 0.6	1			
> 0.6	0.58 [0.35–0.96]			
** Morphologic response**^a^		0.150		**0.033**
1	1		1	
2 or 3	1.44 [0.88–2.37]		1.79 [1.05–3.05]	
** RECIST**_**-15**_^a^		**0.023**		
< -15%	1			
≥ -15%	1.77 [1.09–2.88]			
** TTLD ratio change**^a^		0.511		
< -10%	1			
≥ -10%	1.17 [0.74–1.85]			

Evaluation Criteria in Solid Tumors using a modified cut-off value of -15%

*TTLD* Tumor-to-liver density, *ECOG-PS* Eastern Cooperative Oncology Group – Performance Status, *RECIST*_*-15*_ Response

^a^Recorded at the first restaging

In the univariate analysis, OS was correlated with baseline TTLD ratio (> 0.6 *vs.* ≤ 0.6; HR = 0.55, 95% CI: 0.33, 0.92), RECIST_-15_ value at the first restaging (≥ -15% *vs.* < -15%; HR = 1.79, 95% CI: 1.06, 3.00), and date of metastasis diagnosis (synchronous *vs.* metachronous; HR = 0.52, 95% CI: 0.28–0.99), but not with TTLD ratio at the first restaging and morphologic response. In the multivariate analysis, only the RECIST_-15_ value (≥ -15% *vs.* < -15%; HR = 1.75, 95% CI: 1.05–2.94) and time of metastasis diagnosis (synchronous *vs.* metachronous; HR = 0.53, 95% CI: 0.28–1) remained significant (Table 4).

**Table 4:** Univariate and multivariate Cox models to identify associations between clinical/ radiological factors and OS

	**Univariate analysis (*n* = 79)**		**Multivariate analysis (*n* = 66)**	
	**HR [95%CI]**	***p***	**HR [95%CI]**	***p***
**Age**		0.349		
< 60 years	1			
[60 years; 70 years[	0.98 [0.55–1.77]			
≥ 70 years	1.53 [0.80–2.90]			
**ECOG-PS**		0.195		
0	1			
1	1.40 [0.84–2.35]			
**Primary tumor site**		0.125		
Colon	1			
Rectum	1.57 [0.90–2.74]			
**Metastases**		**0.034**		**0.041**
Synchronous	1		1	
Metachronous	0.52 [0.28–0.99]		0.53 [0.28–1.00]	
**Mean tumor density**				
*** At baseline***		0.065		
≤ 63 HU	1			
> 63 HU	0.62 [0.37–1.03]			
*** At the first restaging***		0.167		
≤ 63 HU	1			
> 63 HU	0.68 [0.39–1.19]			
**TTLD ratio**				
*** At baseline***		**0.024**		
≤ 0.6	1			
> 0.6	0.55 [0.33–0.92]			
*** At the first restaging***		0.064		
≤ 0.6	1			
> 0.6	0.61 [0.36–1.04]			
** Morphologic response**^1^		0.334		
1	1			
2 or 3	1.32 [0.75–2.29]			
** RECIST**_**-15**_^1^		**0.031**		**0.037**
< -15%	1		1	
≥ -15%	1.79 [1.06–3.00]		1.75 [1.05–2.94]	
** TTLD ratio change**^1^		0.663		
< -10%	1			
≥ -10%	1.12 [0.67–1.85]			

*TTLD* Tumor-to-liver density, *ECOG-PS* Eastern Cooperative Oncology Group – Performance, *RECIST*_*-15*_ Response Evaluation Criteria in Solid Tumors using a modified cut-off value of -15%

^1^Recorded at the first restaging

### Correlation between CT and quantitative CEUS parameters

**Fig. 2:**
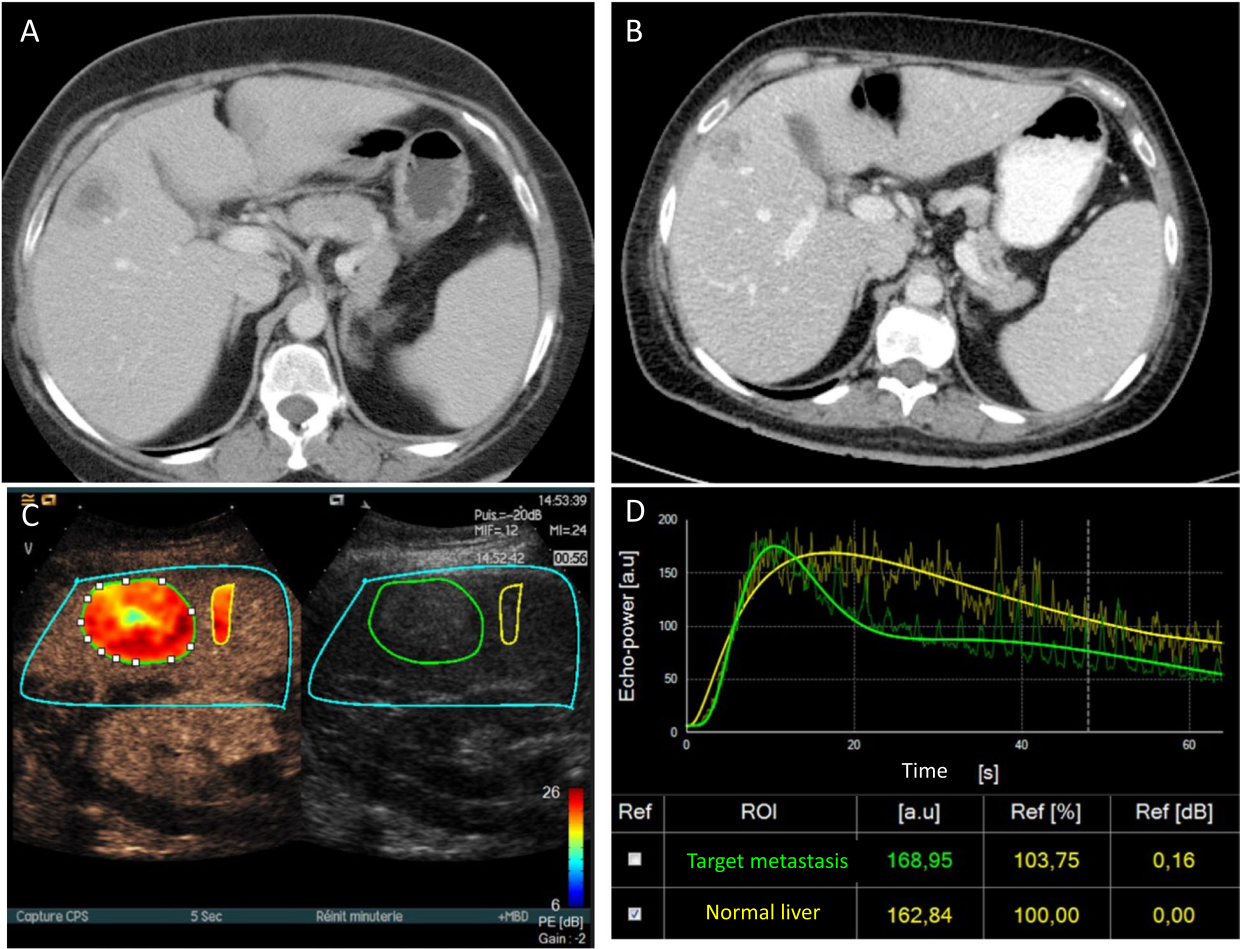
Liver metastasis with a high TTLD ratio with correlative CEUS images and data. Example of CT images acquired during the portal venous phase showing a metastasis with a TTLD ratio > 0.6 at baseline (**A**) and at first restaging (**B**). Late phase correlative CEUS image of the same lesion captured at baseline after SonoVue® injection with ROI drawing around the metastatic lesion delineated in green and the reference normal liver tissue in yellow (**C**). Time-intensity curve of both ROIs with absolute values of peak enhancement and value relative to the healthy liver (in percent, determined by the ratio lesion/reference values *100) (**D**)

When patients were divided in two groups based on the median TTLD ratio at baseline (i.e. 0.6), survival (particularly PFS) was correlated with the TTLD ratio at baseline and at the first restaging. Therefore, quantitative CEUS parameters at D0 and D60 were compared in these two patient groups. At both time points, almost all blood volume parameters were significantly higher in patients with TTLD ratio > 0.6. Among the blood flow parameters, only WiAUC, WiPI and WoR rates (both time points) and mTT (D60) were significantly higher in the TTLD ratio > 0.6 group (Table 5). Fig. 2 illustrates a liver metastasis with a high TTLD ratio before and after treatment on the CT scan and a high peak enhancement measured by CEUS at D0.

**Table 5:** Correlations between TTLD ratio and quantitative CEUS parameters

	Day 0			**Day 60**		
	**TTLD ≤ 0.6**	**TTLD > 0.6**	***p***	**TTLD ≤ 0.6**	**TTLD > 0.6**	***p***
*Blood volume parameters*						
** PE** Missing^1^	36.5 [7.9–217.2] 3	63 [13.6–189.9] 7	**0.007**	30.5 [6.4–238.7] 12	53.9 [14.4–242.0] 10	**0.004**
** WiAUC** Missing^1^	23.8 [3.7–53.0] 3	31.5 [8.1–151.7] 7	**0.034**	21.7 [5.6–56.7] 12	32.1 [11.5–103.2] 10	**0.007**
** WoAUC** Missing^1^	23.7 [3.0–63.3] 10	32.9 [8.1–196.5] 22	**0.053**	16 [5.9–51.2] 22	30.8 [15.0–168.9] 12	**0.009**
** AUC** Missing^1^	27.3 [3.1–56.7] 10	33.8 [9.3–184.2] 22	**0.05**	17.7 [5.8–50.3] 22	31.2 [14.0–146.8] 12	**0.008**
*Blood flow parameters*						
** TTP** Missing^1^	65.8 [32.2–127.0] 3	59.8 [27.9–100] 7	0.65	72.9 [32.0–126.8] 12	63.1 [34.6–96.6] 10	0.22
** RT** Missing^1^	61.1 [26.4–130.6] 3	55.2 [25.3–109.2] 7	0.377	66.5 [25.5–120.4] 12	69.5 [31.1–103.3] 10	0.445
** FT** Missing^1^	61.9 [20.2–249.9] 10	56.8 [12.8–156.5] 22	0.815	70.2 [23.3–166.9] 22	53.2 [26.6–121.1] 12	0.556
** mTT** Missing^1^	78.8 [3.3–288.1] 3	62.1 [14.7–152.5] 7	0.521	88.2[14.2–425.5] 12	45.4 [7.0–425.5] 10	**0.036**
** WiR** Missing^1^	55.5 [9.8–642.6] 3	122.4 [19.3533.7] 7	**0.018**	41.1 [6.5–746.6] 12	105 [19.5–736.2] 10	**0.009**
** WoR** Missing^1^	55.3 [5.8–399.7] 10	112.4 [24.6–478.0] 22	**0.053**	36.9 [5.7–265.7] 22	109.8 [18.1–919.9] 12	**0.019**
** WiPI** Missing^1^	36.8 [8.3–200.3] 3	61.8 [13.1–170. 4] 7	**0.008**	32.6 [6.3–222.6] 12	54.6 [15.6–237.4] 10	**0.005**

All values are relative to the healthy liver values (in percentage, determined as the lesion/reference value ratio *100), except^1^ for missing data reported as numbers

*PE* Peak enhancement, *WiAUC* Wash-in area under the curve, *WoAUC* Wash-out area under the curve, *AUC* total area under the curve (= WiAUC + WoAUC), *TTP* Time to peak, *RT* Rising time, *FT* Fall time, *mTT* mean transit time, *WiR* Wash-in rate, *WoR* Wash-out rate, *WiPI* Wash-in Perfusion Index

## Discussion

Our study demonstrates that alternative radiological parameters may provide useful prognostic information in patients with CRLM receiving bevacizumab-based first-line therapy. High TTLD ratio was independently associated with better outcome and may reflect a tumor vasculature that is more likely to benefit from anti-angiogenic therapies, as suggested by its correlation with perfusion parameters quantified by CEUS.

More than 15 years after bevacizumab implementation for the routine management of metastatic colorectal cancer, biomarkers are still missing to improve patient selection before treatment and to assess its specific efficacy during treatment. Several circulating and tissue components implicated in angiogenesis have been evaluated, including VEGF. However, to date, they have only shown a prognostic impact and have not been integrated into the “clinical decision making” [11, 21–24]. This can be explained by different reasons. First, angiogenesis mechanisms are very complex and involve a large number of factors that are difficult to address holistically. In addition, bevacizumab is systematically associated with cytotoxic chemotherapy, thus preventing the study of its specific effect. Moreover, the methods used to measure these markers are often not reproducible and several ancillary studies lacked a control group to assess the predictive value [25]. Therefore, an alternative approach has been to assess directly bevacizumab effect on the tumor neo-vasculature using static imaging, mainly by exploiting additional features from the cross-sectional images obtained during routine CT, or dynamic imaging, mainly using functional imaging methods. Based on this alternative approach, here, we assessed the prognostic value of the TTLD ratio, which can be easily measured on portal phase CECT images with a dedicated software, and of a modified, size-based RECIST criterion [19]. We confirmed that PFS and OS were longer in patients with a decrease in the total sum of target liver lesions > 15%. This is consistent with previous studies showing that an early size shrinkage with a less stringent threshold (15 or 20%) than the one used in the RECIST 1.1 may be a surrogate marker of clinical outcome in patients with metastatic colorectal cancer, including those receiving antiangiogenic chemotherapy [20, 26, 27].

Unlike in our previous study [19], the TTLD ratio change between baseline and the first restaging using a threshold of -10% did not predict patient outcome. However, the TTLD ratio at specific time points was still correlated with survival. Specifically, in patients with a TTLD ratio > 0.6 at baseline and at the first restaging, the risk of progression was significantly reduced at both time points. This correlation remained significant in the multivariate analysis only for the baseline TTLD ratio. We observed the same trend for OS, although it was not significant in the multivariate analysis. We also found differences in quantitative CEUS parameters between patients with TTLD ratio > 0.6 and ≤ 0.6. This may provide a more comprehensive understanding of this candidate surrogate marker. Indeed, blood volume parameters and some of the blood flow parameters were higher in patients with TTLD ratio > 0.6 than with TTLD ≤ 0.6 at baseline and the first restaging. Liver CEUS was developed to better detect and characterize focal lesions through the qualitative assessment of the vascular architecture and phase-specific contrast enhancement relative to the adjacent healthy liver parenchyma. It also allows the quantitative assessment of the solid tumor perfusion using a time-intensity curve that represents the transition of the contrast agent in the region of interest. Moreover, the ultrasound contrast agents used in CEUS remain purely intravascular, unlike those used in CECT and contrast-enhanced MRI. Thus, the data obtained with this technique only model the flow of the contrast microbubbles in the micro- and macro-vasculature of the selected region of interest [28, 29]. Several studies evaluated CEUS parameters in patients with metastatic colorectal cancer treated with bevacizumab-based chemotherapy. Overall, they showed a decrease in the perfusion parameters during the first weeks of therapy, besides some blood flow characteristics. This suggests a bevacizumab anti-angiogenic mechanism of vascular pruning to starve the tumor. These studies also reported that variations in CEUS parameters could sometimes predict survival; however, the optimal cut-offs were inconsistent among studies and pre-treatment parameters were not prognostic [13, 14, 30–34]. Conversely, our study suggests that the patients with tumors that displayed higher perfusion parameters at baseline and that retained a sufficient degree of perfusion, albeit reduced, after treatment have the best prognosis. These results are consistent with the hypothesis made by Jain et al. on the vascular normalizing effect of anti-angiogenic drugs and its prerequisites to alleviate hypoxia [35, 36]. First, a “minimal” tumor vasculature is needed before treatment initiation because the increase of functional vessels cannot overcome the paucity of vessels. Previous studies suggested that tumors with higher baseline microvascular density or higher surrogate markers of tumor microvascular density (such as CT attenuation) are most likely to benefit from anticancer treatments, including bevacizumab [20, 37, 38]. Second, excessive pruning should be avoided during treatment to preserve the benefit of the improved function of normalized vessels, as suggested by the results of an exploratory correlative study of serial biopsies from patients with localized breast cancer receiving bevacizumab-based neoadjuvant chemotherapy [35]. Pre-clinical and clinical studies by Jain’s group confirmed the vasculature-normalizing effect of anti-angiogenic therapies in solid tumors and in non-malignant diseases [39–42]. In a rabbit model of tuberculosis, they repurposed bevacizumab as a host-directed therapy that resulted in the reduction in vessel number and the increase in vessel pericyte coverage and lumen area. This led to improved drug delivery and oxygenation in lung granulomas, which are characteristic of this infection and share abnormal microenvironment features with solid tumors [43].

This study also provided the opportunity to re-evaluate in an independent cohort of patients with CRLM the performance of the morphological response according to the MD Anderson criteria, another recently proposed CT imaging marker to predict the early clinical response to bevacizumab-based treatment. Similarly to previous studies in cohorts of patients with unresectable metastatic colorectal cancer, this marker was independently associated with longer PFS [16–18] but not with OS, as confirmed by Dohan et al*.* in a large cohort of patients treated with first-line FOLFIRI plus bevacizumab [20]. Morphologic criteria are simple to measure, require a short learning curve and show a strong interobserver agreement, especially to accurately identify optimal responders and non-responders [15, 20, 44, 45]. However, they are underused, possibly because of the reluctance to use a subjective method and the limitations of suboptimal CT techniques.

The TTLD ratio is more objective, but has several limitations. First, it provides only a rough and global assessment of the metastases. For example, it does not take into account the heterogeneity of the intra-tumoral vessel distribution and of the inter-tumoral response. Moreover, as the microvascular volume represents only 10% of the whole tumor volume, this ratio also takes into account characteristics of the non-vascular compartment [46]. However, this could be an advantage because it could be used as a surrogate marker for other components of the tumor microenvironment that may positively or negatively influence the treatment efficacy. Future studies should assess the correlation with histological tissue features. In addition, part of the target lesions selected for the CT analysis were not necessarily the same as those selected for the ultrasound analysis. Eventually, currently, the TTLD ratio seems to correlate mainly with microvascular density but it is not indicative of their functionality, unlike dynamic contrast-enhanced MRI and perfusion CT that can assess the capillary permeability.

This study has some limitations, particularly the small sample size that restricted the statistical analysis. However, this is the first study that assessed the correlation of different radiologic biomarkers to better understand bevacizumab activity. Two other limitations are the absence of a control group to assess their predictive value of the bevacizumab activity, and the inclusion of patients who received different chemotherapy regimens, although irinotecan-based chemotherapy was the most frequent.

The identification of predictive biomarkers of anti-angiogenic drug efficacy should remain a priority in the next years. These agents are expected to play a significant role especially in the management of patients with metastatic colorectal cancer. For instance, following the results of the randomized trial SUNLIGHT, bevacizumab is now indicated, in combination with trifluridine-tipiracil (FTD-TPI), in patients with refractory metastatic colorectal cancer. Indeed, Prager et al. demonstrated that bevacizumab addition to FTD-TPI improves OS compared to FTD-TPI alone [47]. Moreover, fruquintinib, a highly selective oral inhibitor of the VEGF receptors 1, 2, and 3, is now introduced in daily practice as it showed significant benefits compared with placebo in heavily pretreated patients in the FRESCO 2 phase III study [48]. However, although the effectiveness of prolonged angiogenic inhibition is confirmed, the therapeutic benefit is limited to only few months and tumor shrinkage is rare. In addition, there are ongoing studies to repurpose bevacizumab and other anti-angiogenic molecules in combination with immunotherapy since combining immune and vascular modulation is a promising way to improve cancer therapy. Indeed, the abnormal tumor angiogenesis also promotes immunosuppressive functions [49]. On the other hand, different immune cells, such as CD8 + and CD4 + T cells and eosinophils, can contribute to vascular normalization [50]. However, so far, this combination has given very contrasting results in gastrointestinal malignancies. For instance, the combination of the immune checkpoint inhibitor (ICI) atezolizumab with bevacizumab has become the standard first-line treatment in advanced hepatocellular carcinoma [51]. Conversely, a recent study showed that the combination of the ICI pembrolizumab with lenvatinib, another VEGF receptor inhibitor, did not improve OS compared to the standard of care in 480 patients with previously treated metastatic colorectal cancer who were unselected for microsatellite instability high/mismatch repair deficiency [52].

We think that imaging approaches have undeniable advantages to identify the best responders to these different strategies. They are non-invasive, repeatable and provide a more comprehensive view of the tumor and its microenvironment compared with blood biomarkers, which are always affected by systemic dilution due to the influence of normal tissues or the need for a multi-analytic approach given the complexity of the studied phenomena. Although our approach may be considered simple, it lays the groundwork for the future development of an optimized radiological marker for vascular or microenvironment normalization. For example, it would be interesting to determine whether the TTLD ratio correlates with the ICI lack of efficacy frequently observed in patients with CRLM [53, 54]. Lastly, significant advances could also come from the development of more sophisticated image analysis systems based on radiomics and artificial intelligence. These systems have recently shown very promising results in patients with gastrointestinal cancer [55, 56].

## Conclusions

In this study, we showed that in patients with CRLM, clinical outcome correlated with early tumor shrinkage, but not with TTLD ratio variations. Nevertheless, the TTLD ratio absolute value, especially at baseline, may predict the efficacy of bevacizumab-based treatment by reflecting tumor microenvironment features that may influence its effect.

## Data Availability

The datasets used and/or analyzed in the current study are available from the corresponding author upon reasonable request.
